# What incentives increase data sharing in health and medical research? A systematic review

**DOI:** 10.1186/s41073-017-0028-9

**Published:** 2017-05-05

**Authors:** Anisa Rowhani-Farid, Michelle Allen, Adrian G. Barnett

**Affiliations:** Australian Centre for Health Services Innovation, Institute of Health and Biomedical Innovation, Queensland University of Technology, 60 Musk Avenue, Kelvin Grove, 4059 Australia

**Keywords:** Incentives, Data sharing, Open data, Meta-research

## Abstract

**Background:**

The foundation of health and medical research is data. Data sharing facilitates the progress of research and strengthens science. Data sharing in research is widely discussed in the literature; however, there are seemingly no evidence-based incentives that promote data sharing.

**Methods:**

A systematic review (registration: 10.17605/OSF.IO/6PZ5E) of the health and medical research literature was used to uncover any evidence-based incentives, with pre- and post-empirical data that examined data sharing rates. We were also interested in quantifying and classifying the number of opinion pieces on the importance of incentives, the number observational studies that analysed data sharing rates and practices, and strategies aimed at increasing data sharing rates.

**Results:**

Only one incentive (using open data badges) has been tested in health and medical research that examined data sharing rates. The number of opinion pieces (*n* = 85) out-weighed the number of article-testing strategies (*n* = 76), and the number of observational studies exceeded them both (*n* = 106).

**Conclusions:**

Given that data is the foundation of evidence-based health and medical research, it is paradoxical that there is only one evidence-based incentive to promote data sharing. More well-designed studies are needed in order to increase the currently low rates of data sharing.

**Electronic supplementary material:**

The online version of this article (doi:10.1186/s41073-017-0028-9) contains supplementary material, which is available to authorized users.

## Rationale

Despite the current shift towards more open data in health and medical research, there are seemingly no evidence-based incentives that increase data sharing. As such, a systematic review was used to verify the lack of evidence-based incentives in this area.

## Objective

This study aims to systematically review the literature to appraise and synthesise scientific research papers that concern incentives that have been tested to increase data sharing in health and medical research.

## Background

### Research waste: hidden data, irreproducible research

The foundation of health and medical research is data—its generation, analysis, re-analysis, verification, and sharing [1]. Data sharing is a key part of the movement towards science that is open, where data is easily accessible, intelligible, reproducible, replicable, and verifiable [2]. Data sharing is defined here as making raw research data available in an open data depository, and includes controlled access where data is made available upon request which may be required due to legal or ethical reasons. Despite the wide-scale benefits of data sharing such as addressing global public health emergencies, it is yet to become common research practice. For instance, the severe acute respiratory syndrome (SARS) disease was controlled only 4 months after its emergence by a World Health Organization-coordinated effort based on extensive data sharing [3]. Likewise, the researchers working on the Ebola outbreak have recently committed to work openly in outbreaks to honour the memory of their colleagues who died at the forefront of the Ebola outbreak, and to ensure that no future epidemic is as devastating [4]. Notwithstanding these benefits, numerous studies have demonstrated low rates of data sharing in health and medical research, with the leading journal the British Medical Journal (BMJ) having a rate as low as 4.5% [5] and biomedical journal articles 0% [6]. There are of course legitimate reasons to withhold data, such as the concern about patient privacy, and the requirement for patient consent for sharing [7].

With 85% of the world’s spending on health and medical research, an estimated $170 billion, wasted every year, it is clear that the scientific community is in crisis, leading to questions about the veracity of scientific knowledge [8]. Data sharing and openness in scientific research should be fundamental to the philosophy of how scientific knowledge is generated. Thomas Kuhn introduced the concept of paradigm shifts that arise from a scientific crisis. The paradigm shift before us today is from closed, hidden science to open science and data sharing [9]. Sharing scientific data will allow for data verification and re-analysis, and for testing new hypotheses. Open data reduces research waste in terms of time, costs, and participant burden, and in turn, strengthens scientific knowledge by ensuring research integrity [5, 10].

The many current problems in health and medical research have led to the emergence of a new field, meta-research, which is concerned with improving research practices [2]. Meta-research has five sub-themes with ‘reproducibility’ and ‘incentives’ as two of the themes [2]. Reproducibility is concerned with the verification of research findings, which can be achieved through the sharing of data and methods [2]. Incentives is concerned with rewarding researchers, which includes incentives to share their data and methods [2]. We were interested in how researchers are incentivised to openly share their raw data, thus combining two sub-themes of meta-research.

### Research waste: historical barriers

Historically, it has not been common practice for the content of a research article to include access to the raw data from scientific experiments [11]. This flaw, created by technological limitations among others, has hindered the progress of scientific knowledge [5]. However, we can no longer blame technology for outdated research practices. There are many data depositories which allow researchers to easily share their data using a citable DOI. There have also been many recent policies and frameworks to encourage openness in research [7]. Yet, uptake in health and medicine is low and what is lacking, it appears, are rewards that incentivize researchers to share their data [11]. Incentives are defined here as rewards that are given to researchers if they participate in sharing their raw scientific data [12].

## Research design and methodology

The Queensland University of Technology (QUT) Library staff assisted in developing a rigorous and clearly documented methodology for both the search strategy and the selection of studies. The aim was to minimise bias by documenting the search process and the decisions made to allow the review to be reproduced and updated.

The Cochrane Handbook for Systematic Reviews was used as a guide for this systematic review: http://handbook.cochrane.org/. The EQUATOR Network Additional file 1: PRISMA (2009) Checklist [13] was used to ensure good practice as well as accurate reporting.

Three systematic review registries (Prospero, Joanna Briggs Institute, and Cochrane) were checked to ensure our proposed systematic review had not already been done. Our systematic review protocol was registered at the Open Science Framework on 1 August 2016 (10.17605/OSF.IO/6PZ5E).

### Inclusion criteria

#### Types of documents

This review considered published journal articles with empirical data that trialed any incentive to increase data sharing in health and medical research.

#### Types of data

Articles must have tested an incentive that could increase data sharing in health and medical research. For the purposes of this review, health and medical research data is defined as any raw data that has been generated through research from a health and medical facility, institute or organisation.

Incentives are defined here as ‘a benefit, reward, or cost that motivates an […] action’. This was based on the definition of incentives in economics, which groups incentives into four categories: financial, moral, natural, and coercive [14].

#### Types of measures

The review included any paper with empirical data on sharing that compared an intervention and control, which used a clear research design (including randomised and non-randomised designs). The types of measures included are the percent of datasets shared, or the number of datasets shared, or the relative ratio of data sharing.

### Exclusion Criteria

This review excluded the following, but still classified these excluded papers by field:

- all editorial and opinion pieces that only discuss strategies to increase data sharing without trialling them.
- strategies that do not involve incentives, e.g., education seminars, change in a data sharing policy or some other policy, access to data management tools and managers.
- observational studies that describe data sharing patterns.

### Search Strategy

This search strategy was designed to access published articles through the following steps:

1. (((“open science” OR “open data” OR “data sharing”) AND (incentive* OR motivation* OR reward* OR barrier*)))
2.
  **Table:** 

Database Health/Medical	Search
PubMed	((“open science” OR “open data” OR “data sharing”) AND (incentive* OR motivation* OR reward* OR barrier*))
EMBASE	((“open science” OR “open data” OR “data sharing”) AND (incentive* OR motivation* OR reward* OR barrier*))
CINAHL	((“open science” OR “open data” OR “data sharing”) AND (incentive* OR motivation* OR reward* OR barrier*))
Multi-disciplinary databases	Search
Scopus	((“open science” OR “open data” OR “data sharing”) AND (incentive* OR motivation* OR reward* OR barrier*))
Web of Science	((“open science” OR “open data” OR “data sharing”) AND (incentive* OR motivation* OR reward* OR barrier*))
3. Relevant articles that did not appear in the database search but were known to the reviewers were hand-picked and extracted into EndNote.

### Process of selecting and evaluating articles

Two reviewers, ARF and MA, screened the titles of the articles and based on the inclusion and exclusion criteria, extracted them into EndNote. Duplicates were removed.

The reviewers independently screened the extracted article titles and abstracts based on the inclusion and exclusion criteria and categorised them into five groups:

1. Incentives
2. Other strategies
3. Opinion pieces
4. Observational studies
5. Irrelevant

ARF read the titles and abstracts of all extracted articles and MA verified her findings by reading a random sample of 30%. Discrepancies between the two reviewers were approximately 10%, however these were relatively minor and resolved through discussion of the scope of each of the categories. For instance, a research paper outlined the introduction of a data system, one reviewer classified it as an observational study, but after discussion it was agreed that it was a strategy article as its objective was to increase data sharing rates rather than observing data sharing patterns.

### Process of extracting relevant information

The two reviewers independently read eligible documents and extracted data sharing incentives in health and medical research. Both reviewers were agnostic regarding the types of incentives to look for. The final list of incentives was determined and agreed on by all authors [11].

### Data synthesis

Individual incentives were grouped into research fields. A qualitative description of each incentive was presented.

Based on our prior experience of the literature, the research fields and sub-fields for classification were:

- Health and medical research
  i. Psychology
  ii. Genetics
  iii. Other (health/medical)
  b. Non-health and medical research
    i. Information technology
    ii. Ecology
    iii. Astronomy
    iv. Other (non-health/medical)

The other article–strategies, opinion pieces, and observational studies were also grouped into the same research fields.

## Results

The database searches found 1415 articles, 1039 of which met the inclusion criteria based on assessment of titles and abstracts and were exported into EndNote. After automatically removing duplicates, 670 articles remained and after manually removing the remainder of the duplicates, 586 articles remained.

586 titles and abstracts were read and categorised based on the above inclusion and exclusion criteria. One study was hand-picked as it met the inclusion criteria, bringing the total number of extracted articles to 587. After screening titles and abstracts, nine articles were classified under incentives in health and medical research. These articles were then read in full, and one of them was judged as an incentive that satisfied the inclusion criteria.

**Fig. 1:**
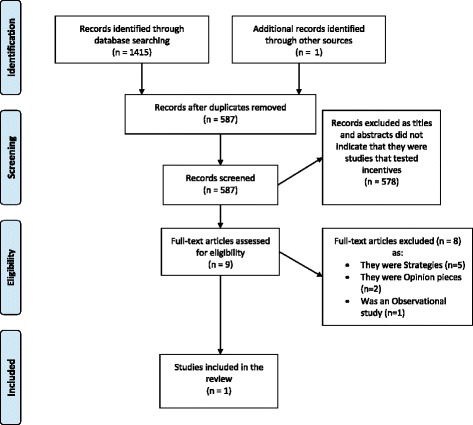
PRISMA [13] Flow Chart: systematic selection of studies that have tested incentives to increase data sharing rates in health and medical research from the literature

The PRISMA [13] flow chart that outlines the journey of the articles from identification to inclusion is in Fig. 1. The categorisation of all 587 articles into the sub-fields and article type is in Table 1.

**Table 1:** Categorisation of all screened articles into sub-fields and article type

Research fields and sub-fields	Article type				
	Incentives	Strategies	Opinion Pieces	Observational Studies	Total: field of studies
*Health and Medical Research*					
Psychology	1	2	4	1	8
Genetics	0	16	12	17	45
Other	0	58	69	88	215
Total: health and medical research	*1*	*76*	*85*	*106*	*268*
*Non-Health and Medical Research*					
Astronomy	0	0	0	1	1
Ecology	0	5	11	8	24
Information Technology	0	38	26	28	92
Other	0	46	52	87	185
Total: non-health and medical research	*0*	*89*	*89*	*124*	*302*
Total: type of studies	*1*	*149*	*174*	*230*	*570*

A review of the reference list for the one included intervention was undertaken [15]. The titles and abstracts of the full reference list of this study (23 papers) and those that cited the study (5 papers) were read, but none met the inclusion criteria of this systematic review.

17 articles were irrelevant, bringing the total number of screened articles to 570. The distribution of articles across type of study was similar for both health and medical research and non-health and medical research (Table 1). Observational studies were the most common type (n = 106, n = 124), then opinion pieces (*n* = 85, *n* = 89), then articles testing strategies (*n* = 76, *n* = 89), and articles testing incentives were uncommon (*n* = 1, *n* = 0).

### Observational studies about data sharing in health and medical research

These articles did not fit the inclusion criteria, but based on the abstracts they were mostly concerned with observing data sharing patterns in the health and medical research community, using quantitative and qualitative methods. The motivation behind these studies was often to identify the barriers and benefits to data sharing in health and medical research. For instance, Federer et al. (2015) conducted a survey to investigate the differences in experiences with and perceptions about sharing data, as well as barriers to sharing among clinical and basic science researchers [16].

### Opinion pieces about data sharing in health and medical research

These articles also did not fit the inclusion criteria, but based on the abstracts they were opinion and editorial pieces that discussed the importance and benefits of data sharing and also outlined the lack of incentives for researchers to share data.

### Main results: incentives in health and medical research

#### Badges

Open data and open material badges were created by the Center of Open Science and were tested at the journal *Psychological Science* [15]. In January 2014, the journal adopted badges to acknowledge open data, open materials and preregistration of research if published [15]. A Badges Committee at the Centre of Open Science outlined what it meant to have ‘open data’ and ‘open materials’ and the journal editorial team awarded badges to those authors who voluntarily applied for them upon article acceptance and proved that they met the criteria [15]. The criteria to earn an open data or open materials badge involved making all digitally sharable data and materials available in an open data repository [15]. Badges greatly increased the reported open data rate at the journal from 1.5% in the first half of 2012 (start point) to 39.4% in the first half of 2015 (end point) [15].

#### Limitations

A limitation of the badge study was that it did not use a randomized parallel group design; notwithstanding, it was the only incentive that was tested in the health and medical research community, with pre- and post-incentive empirical data [15]. The pre- and post-design of the study makes it vulnerable to other policy changes over time, such as a change from a government funding agency like the recent Statement on Data Sharing from the Australian National Health and Medical Research Council [17]. However, the Kidwell et al. study addressed this concern with contemporary control journals. A limitation of the badge scheme was that even with badges, the accessibility, correctness, usability, and completeness of the shared data and materials was not 100%, which was attributable to gaps in specifications for earning badges. In late 2015, the Center for Open Science Badges Committee considered provisions for situations in which the data or materials for which a badge was issued somehow disappear from public view and how adherence to badge specifications can be improved by providing easy procedures for editors/journal staff to validate data and material availability before issuing a badge, and by providing community guidelines for validation and enforcement [15].

### Incentives in non-health and medical research

Of the non-health/medical incentives, seven were categorised as information technology, and nine as other. Upon reading the full text, all the 16 non-health/medical incentives were proposed incentives or strategies as opposed to tested incentives with comparative data.

### Strategies to increase data sharing in health and medical research

Given that the systematic review found only one *incentive*, we classified the data sharing *strategies* tested in the health and medical research community. Seventy-six articles were classified under ‘strategies’ and Table 2 shows the further classification into categories based on a secondary screening of titles and abstracts. The articles are grouped by whether they presented any data, descriptive, or empirical.

**Table 2:** Categorisation of the 76 data sharing strategy articles

Sub-theme of health and medical research	Category (numbers)	Empirical or descriptive data	None or little empirical or descriptive data
Psychology	Data system **(** *2* **)**	*2*[33, 34]	
Genetics	Data system **(** *14* **)**	*12*[19, 35–45]	*2*[46, 47]
	Collaboration and data system **(** *1* **)**		*1*[25]
	Collaboration **(** *1* **)**		*1*[48]
Other (health and medical research)	Data system **(** *41* **)**	*35* [19–21, 49–80]	*6* [81–86]
	Collaboration and data system **(** *7* **)**	*7* [22, 23, 87–91]	
	Collaboration **(** *6* **)**	*3*[24, 92, 93]	*3* [94–96]
	Policy **(** *3* **)**	*2*[26, 97]	*1*[98]
	Campaign **(** *1* **)**		*1*[27]

The majority, 57/76, of strategies were technological strategies such as the introduction of a data system to manage and store scientific data. Seven of the 76 strategies concerned encouraging collaboration among research bodies to increase data sharing. Eight were a combination of collaboration across consortia and the introduction of a technological system. Three had a data sharing policy as the strategy but did not test the effectiveness of the policy, but two of them reported descriptive data from their experience in implementing the policy. One strategy was an open data campaign.

Below we give some examples of the strategies used to promote data sharing.

### Strategies in health and medical research: data systems

#### Dataset linkage—attribution

Two articles discussed an incentive system for human genomic data and data from rare diseases, namely, microattribution and nanopublication—the linkage of data to their contributors. However, the articles only discussed the models and did not present empirical data [18, 19].

Another article discussed the OpenfMRI project that aims to provide the neuroimaging community with a resource to support open sharing of fMRI data [20]. In 2013, the OpenfMRI database had 18 full datasets from seven different laboratories and in October 2016, the database had 55 datasets openly available (https://openfmri.org/dataset/). The authors identified credit as a barrier towards sharing data and so incorporated attribution into the OpenfMRI website where a dataset is linked to the publication and the list of investigators involved in collecting the data [20].

#### Electronic laboratory notebooks

An article discussed open source drug discovery and outlined its experience with two projects, the praziquantel (PZG) project and the Open Source Malaria project [21]. The article did not have pre- and post-strategy data. The authors discussed the constituent elements of an open research approach to drug discovery, such as the introduction of an electronic lab notebook that allows the deposition of all primary data as well as data management and coordination tools that enhances community input [21]. The article describes the benefits and successes of the open projects and outlines how their uptake needs to be incentivised in the scientific community [21].

### Strategies in health and medical research: collaboration and data system

An article discussed the development of the Collaboratory for MS3D (C-MS3D), an integrated knowledge environment that unites structural biologists working in the area of mass spectrometric-based methods for the analysis of tertiary and quaternary macromolecular structures (MS3D) [22]. C-MS3D is a web-portal designed to provide collaborators with a shared work environment that integrates data storage and management with data analysis tools [22]. The goal is not only to provide a common data sharing and archiving system, but also to assist in the building of new collaborations and to spur the development of new tools and technologies [22].

#### Attribution

One article outlined the collaborative efforts of the Global Alzheimer’s Association Interactive Network (GAAIN) to consolidate the efforts of independent Alzheimer’s disease data repositories around the world with the goals of revealing more insights into the causes of Alzheimer’s disease, improving treatments, and designing preventative measures that delay the onset of physical symptoms [23]. In 2016, they had registered 55 data repositories from around the world with over 25,000 subjects using GAAIN’s search interfaces [23]. The methodology employed by GAAIN to motivate participants to voluntarily join its federation is by providing incentives: data collected by its data partners are advertised, as well as the identity of the data partners, including their logos and URL links, on each GAAIN search page [23]. GAIIN attributes its success in registering 55 data repositories to date to these incentives which provide opportunities for groups to increase their public visibility while retaining control of their data, making the relationship between GAIIN and its partners mutually beneficial [23]. This study did not have pre- and post-strategy empirical data, but described the importance of incentives in motivating researchers to share their data with others [23].

### Strategies in health and medical research: collaboration

An article described how data sharing in computational neuroscience was fostered through a collaborative workshop that brought together experimental and theoretical neuroscientists, computer scientists, legal experts, and governmental observers [24]. This workshop guided the development of new funding to support data sharing in computational neuroscience, and considered a conceptual framework that would direct the data sharing movement in computational neuroscience [24]. The workshop also unveiled the impediments to data sharing and outlined the lack of an established mechanism to provide credit for data sharing as a concern [24]. A recommendation was that dataset usage statistics and other user feedback be used as important measures of credit [24].

One article addressed the need to facilitate a culture of responsible and effective sharing of cancer genome data through the establishment of the Global Alliance for Genomic Health (GA4GH) in 2013 [25]. The collaborative body unpacked the challenges with sharing cancer genomic data as well as the potential solutions [25]. The GA4GH developed an ethical and legal framework for action with the successful fostering of an international ‘coalition of the willing’ to deliver a powerful, globally accessible clinic-genomic platform that supports data-driven advances for patients and societies [25].

### Strategies in health and medical research: policy

An article discussed the efforts of the Wellcome Trust Sanger Institute to develop and implement an institute-wide data sharing policy [26]. The article outlined that successful policy implementation depends on working out detailed requirements (guidance), devoting efforts and resources to alleviate disincentives (facilitation), instituting monitoring processes (oversight), and leadership [26]. The topic of disincentives (facilitation) included concerns about lack of credit [26]. They propose that cultural barriers to data sharing continue to exist and that it is important to align the reward system to ensure that scientists sharing data are acknowledged/cited and that data sharing is credited in research assessment exercises and grant career reviews [26].

### Strategies in health and medical research: campaign

One intervention was an open data campaign which was included in the review via an open letter in June 2014 from the AllTrials campaign to the director of the European Medicines Agency to remove barriers to accessing clinical trial data [27]. The AllTrials campaign is supported by more than 78,000 people and 470 organisations worldwide [27]. This letter contributed to the European Medicines Agency publishing the clinical reports underpinning market authorization requests for new drugs, which was part of a more proactive policy on transparency that applied to all centralized marketing authorisations submitted after 1 January 2015 [27]. The adoption of this policy was a significant step in ensuring transparency of health and medical research in Europe [27].

## Discussion

This systematic review verified that there are few evidence-based incentives for data sharing in health and medical research. The irony is that we live in an evidence-based world, which is built upon the availability of raw data, but we hardly have any evidence to demonstrate what will motivate researchers to share data. To date, open data badges are the only tested incentive. Badges are an effective signal and incentive for open practices and journals can offer them to authors who are willing and able to meet criteria to earn an open data and open material badge [15].

It is interesting to note the great number of opinion pieces (*n* = 85) on the importance of developing incentives for researchers, which outnumbered the number of articles that tested strategies to increase data sharing rates (*n* = 76). ‘Opinion pieces’ are mutually exclusive from ‘strategies’ as the former is concerned with discussing possible strategies and incentives and the latter tests the ideas and strategies and provides evidence of what works or does not work. These strategies included: the introduction of data systems such as electronic laboratory notebooks and databases for data deposition that incorporated a system of credit through data linkage; collaboration across consortia that also introduce data systems that also use data attribution as an incentive; collaboration across consortia through workshops and development of frameworks for data sharing; implementation of data sharing policies; and campaigns to promote data sharing. These strategies discussed the requirement of introducing rewards to increase data sharing rates and the only form of incentive used was via data attribution and advertising on websites. Studies that test the effectiveness of attribution and advertising as a form of credit are necessary.

In light of the small number of studies, we see a clear need for studies to design and test incentives that would motivate researchers to share data. Organisations are promoting the development of incentives to reduce research waste. In late 2016, the Cochrane and the REWARD alliance combined to create the annual Cochrane-REWARD prize for reducing waste in research. The monetary prize is awarded to ‘any person or organisation that has tested and implemented strategies to reduce waste in one of the five stages of research production [question selection, study design, research conduct, publication, and reporting] in the area of health’. This prize is an example of an incentive for researchers to design studies or implement policies that reduce research waste; it will be interesting to see the impact of this initiative [28].

Another endeavour in the area of developing incentives and rewards for researchers is the convening in early 2017 of a group of leaders from the USA and Europe from academia, government, journals, funders, and the press to help develop new models for academic promotion and professional incentives that would promote the highest quality science, organised by the Meta-Research Innovation Center at Stanford (METRICS). The focus will be on designing practical actions that embody principles that this community has embraced, while also recognizing that the effect of any such policies will need empirical evaluation.

While the systematic barriers to widespread data sharing are being addressed through the general shift towards more openness in research, the conversation on data sharing includes an alternative view where users of shared data are called ‘research parasites’ who ‘steal from research productivity’ and who are ‘taking over’ [29, 30]. There is also some questioning of whether data sharing is worth the effort [30]. These points, however, are contrary to the purpose of sharing data, which is to progress science as a body of knowledge and to make the research process more robust and verifiable [5, 30].

## Limitations

A limitation of this systematic review is that we did not search the grey literature (materials and research produced by organizations outside of the traditional commercial or academic publishing and distribution channels). This review could be perceived as having a narrow design, given that we anticipated a lack of evidence-based incentives for data sharing in health and medical research, hence making the topic of this systematic review too simple. However, we could not be sure that there were no incentives and the recent paper by Lund and colleagues (2016) emphasises the importance of conducting systematic reviews prior to designing interventions in order to avoid adding to the already large issue of research waste [31].

## Conclusions

The current meta-research discourse outlines the numerous benefits of openness in research: verification of research findings, progressing health and medicine, gaining new insights from re-analyses, reducing research waste, increasing research value, and promoting research transparency. However, this systematic review of the literature has uncovered a lack of evidence-based incentives for researchers to share data, which is ironic in an evidence-based world. The open data badge is the only tested incentive that motivated researchers to share data [15]. This low-cost incentive could be adopted by journals and added to the reward system to promote reproducible and sharable research [15, 32]. Other incentives like attribution require empirical data. Instead of evidence-based incentives, the literature is full of opinion pieces that emphasize the lack of incentives for researchers to share data, outweighing the number of strategies that aim to increase data sharing rates in health and medicine. Observational studies that identify data sharing patterns and barriers are also plentiful, and whilst these studies can provide useful background knowledge, they do not provide good evidence of what can be done to increase data sharing.

## Additional file

Additional file 1: PRISMA (2009) Checklist. (DOC 62 kb)

## Abbreviations

AGB: Adrian Gerard Barnett
ARF: Anisa Rowhani-Farid
C-MS3D: Collaboratory for MS3D
GA4GH: Global Alliance for Genomic Health
GAAIN: Global Alzheimer’s Association Interactive Network
MA: Michelle Allen
METRICS: Meta-Research Innovation Center at Stanford
PRISMA: Preferred Reporting Items for Systematic Reviews and Meta-Analyses
PZG: Praziquantel
QUT: Queensland University of Technology
REWARD: Reduce research Waste and Reward Diligence
WTSI: Wellcome Trust Sanger Institute
