# Erratum to: What incentives increase data sharing in health and medical research? A systematic review

**DOI:** 10.1186/s41073-017-0034-y

**Published:** 2017-05-23

**Authors:** Anisa Rowhani-Farid, Michelle Allen, Adrian G. Barnett

**Affiliations:** Australian Centre for Health Services Innovation, Institute of Health and Biomedical Innovation, Queensland University of Technology, 60 Musk Avenue, Kelvin Grove, 4059 Australia

## Erratum

In the original publication of this article [1] there has been an error in the display of table 1. In this Erratum the erroneous table 1 (Table 1 in this Erratum) and the correct table 1 (Table 2 in this Erratum) are published. The original publication of this article has been updated. The publisher apologizes to the readers and authors for the oversight.

**Table 1:** Original version of Table 1 as published on 5 May 2017

Research fields and sub-fields	Article type				
*Health and medical research*					
Psychology	1	2	4	1	8
Genetics	0	16	12	17	45
Other	0	58	69	88	215
Total: health and medical research	*1*	*76*	*85*	*106*	*268*
*Non-health and medical research*					
Astronomy	0	0	0	1	1
Ecology	0	5	11	8	24
Information technology	0	38	26	28	92
Other	0	46	52	87	185
Total: non-health and medical research	*0*	*89*	*89*	*124*	*302*
Total: type of studies	*1*	*149*	*174*	*230*	*570*

**Table 2:** Updated version of Table 1, the missing headings have been added

Research fields and sub-fields	Article type				
	Incentives	Strategies	Opinion Pieces	Observational Studies	Total: field of studies
*Health and Medical Research*					
Psychology	1	2	4	1	8
Genetics	0	16	12	17	45
Other	0	58	69	88	215
Total: health and medical research	1	76	85	106	268
*Non-Health and Medical Research*					
Astronomy	0	0	0	1	1
Ecology	0	5	11	8	24
Information Technology	0	38	26	28	92
Other	0	46	52	87	185
Total: non-health and medical research	0	89	89	124	302
Total: type of studies	1	149	174	230	570
